# Assessing muscle morphology and mechanics: one protocol does not fit all

**DOI:** 10.1007/s10974-026-09721-6

**Published:** 2026-01-27

**Authors:** R. W. P. Kissane

**Affiliations:** Department of Musculoskeletal & Ageing Science, University of Liverpool, The William Henry Duncan Building, 6 West Derby Street, L7 8TX Liverpool, UK

In the recent study by Cesanelli et al. (2025a) they discuss the importance of myostatin for the morphological and contractile adaptations of skeletal muscle to functional overload. Understanding the architectural and contractile function of skeletal muscle in response to adaptive and pathophysiological remodelling is a complex approach to execute well. While many of the conclusions made in this manuscript are likely valid, there are several contentious methodological issues that I would like to highlight as areas of consideration and refinement for those undertaking similar types of experiments in the future.

Firstly, addressing muscle morphology/architecture, which are umbrella terms typically used to include muscle mass, fibre length, pennation angle and physiological cross-sectional area (PCSA). These factors are critical determinants for the ability of skeletal muscle to generate force and power. Accurately measuring muscle fibre length is particularly important as it underpins the calculation of PCSA, and subsequently the estimated ability of this muscle to generate force (Charles et al. 2022). The authors unfortunately have neglected to measure fibre length, rather using an estimate of fibre length by multiplying the optimal length (L_0_) by 0.7, taken from (Brooks and Faulkner 1988). While this is standard across the field, it is troublesome for two reasons; firstly there are no data on the relative fibre lengths of the transgenic mouse line used BEH^++^ or BEH^cc^ which may have different relative fibre lengths, with values in the literature for the mouse soleus ranging from 0.48 to 0.85 (Askew and Marsh 1997; Charles et al. 2016) across different mice strains. Secondly, the functional overload intervention is thought to promote sarcomerogenesis (serial additions of sarcomeres) which will likely have a direct impact on fibre length (Zöllner et al. 2012) and shift the relative muscle to fibre length ratio. The assumptions here for estimating PCSA has knock on effects to the estimates of specific force and stress (measured here), which should be calculated using measured fibre length. Further, having an accurate measure of muscle fibre length is essential to describe any lengthening/shortening velocity relationships.

The second methodological concern relates to the eccentric contraction protocol used here, and elsewhere (Baltusnikas et al. 2015; Cesanelli et al. 2025b). This approach might seem like an efficient approach to derive numerous contractile metrics from a single stretch protocol, however I believe this protocol to be fundamentally flawed and should not be used to make comparisons across experimental groups. The eccentric (active muscle lengthening) force response is a dynamic phenomenon comprising a fast phase-1 response (cross-bridge dependent) and a slow phase-2 (non-cross bridge serial elastic elements dependent) response which are highly velocity dependent (Kissane and Askew 2024). The derived measures in this manuscript for peak eccentric force, specific eccentric force, stiffness and tangent modulus are arguably incomparable across the presented groups for several reasons. Firstly, the authors have not presented the active force-length relationship for the different groups. With the aforementioned potential for sarcomerogenesis and inter-strain differences in fibre length there is a large possibility that the force-lengths relationship differ among experimental groups. Therefore, the starting length of the eccentric ramp used in this experiment will likely have inconsistent levels of overlapping actin-myosin filaments, which may significantly impact the rate of force rise during eccentric loading (Krylow and Sandercock 1997) and subsequently the peak eccentric force measure described here. Secondly, the rate of force development during the phase-1 and phase-2 components present with a significant velocity dependence, maintained when normalised to maximum shortening velocity (Kissane and Askew 2024). Cesanelli et al. stretched each sample at a single velocity (1.5 fibre lengths per second), a value that cannot be meaningfully related to the maximum shortening velocity of these muscles, which are likely to differ among each of the groups. Given the sensitivity of the rate of force development (or stiffness) of both the phase-1 (referred to as the early phase by the authors) and phase-2 (referred to as the late phase by the authors) to velocity, I believe the use of a single lengthening velocity to be largely uninformative and not comparable.

Here I have detailed a short list of considerations for those in future who attempt to characterise muscle mechanics; (1) fibre length quantification should be almost a standard internal check across mouse strains, given its importance in quantifying PCSA, normalising muscle stress, and shortening/lengthening velocities. It is a reasonably simple method (Kissane et al. 2018; Schachner et al. 2024) to generate critical data that underpins much of muscle mechanics. (2) Almost every muscle mechanical experiment should begin with a force-length relationship to optimise muscle lengths and determine L_0_. This simple protocol allows the initial overview of both passive and active elements with for very little effort. Further, this simple optimisation step generates twitches that allow the reporting of twitch rise/relaxation times, proving indirect information on calcium handling kinetics (e.g. the ability of release and re-uptake of Ca^2+^ ions by the sarcoplasmic reticulum) (Baylor and Hollingworth 2003). These data are all valuable and essentially generated during the optimisation for the next step. (3) As discussed, a single absolute velocity stretch/shortening protocols are generally not comparable. It is more informative to comprehensively characterise the shortening and lengthening relationship across a range of velocities (i.e. determine V_max_, and lengthen incrementally up to this velocity) to understand the mechanisms of decline in response to pathophysiological remodelling or adaptive remodelling (Tahir et al. 2020; Kissane and Askew 2024).

## Data Availability

No datasets were generated or analysed during the current study.
